# SMAD3 directly regulates cell cycle genes to maintain arrest in granulosa cells of mouse primordial follicles

**DOI:** 10.1038/s41598-019-42878-4

**Published:** 2019-04-24

**Authors:** Sofia Granados-Aparici, Kate Hardy, Stephen Franks, Isam B. Sharum, Sarah L. Waite, Mark A. Fenwick

**Affiliations:** 1 Academic Unit of Reproductive and Developmental Medicine, Department of Oncology and Metabolism, University of Sheffield, Sheffield, S10 2SF United Kingdom; 2 Present Address: Department of Obstetrics and Gynecology, McGill University Health Centre, Montreal, Canada; 3 Institute of Reproductive and Developmental Biology, Imperial College London, Hammersmith Hospital, Du Cane Road, London, W12 0NN United Kingdom; 4 Present Address: Department of Surgery and Theriogenology, College of Veterinary Medicine, University of Mosul, Mosul, Iraq

**Keywords:** Reproductive biology, TOR signalling

## Abstract

Primordial follicles, consisting of granulosa cell (GC)-enveloped oocytes are maintained in a state of developmental arrest until activated to grow. The mechanism that operates to maintain this arrested state in GCs is currently unknown. Here, we show the TGFβ-activated transcription factor SMAD3 is expressed in primordial GC nuclei alongside the cell cycle proteins, cyclin D2 (CCND2) and P27. Using neonatal C57/Bl6 mouse ovaries densely populated with primordial follicles, CCND2 protein co-localised and was detected in complex with P27 by immunofluorescence and co-immunoprecipitation, respectively. In the same tissue, SMAD3 co-precipitated with DNA sequences upstream of *Ccnd2* and *Myc* transcription start sites implicating both as direct SMAD3 targets. In older ovaries follicle growth was associated with nuclear exclusion of SMAD3 and reduced P27 and CCND2 in GCs, alongside elevated *Myc* expression. Brief (2 H) exposure of neonatal ovaries to TGFβ1 (10 ng/ml) *in vitro* led to immediate dissociation of SMAD3 from the *Ccnd2* and *Myc* promoters. This coincided with elevated *Myc* and phospho-S6, an indicator of mTOR signalling, followed by a small increase in mean primordial GC number after 48 H. These findings highlight a concentration-dependent role for TGFβ signalling in the maintenance and activation of primordial follicles, through SMAD-dependent and independent signalling pathways, respectively.

## Introduction

Primordial follicles, each consisting of a central oocyte surrounded by a single layer of supporting granulosa cells (GCs) are held in a relative state of developmental arrest until activated to grow^1–3^. Maintenance and regulation of the arrested state is fundamental for ensuring that a steady supply of oocytes is available for ovulation throughout the reproductive life time in most mammals^4,5^; however, the molecular mechanisms that operate to maintain this quiescent phenotype are currently unclear.

We recently showed that in the mouse ovary, the TGFβ-mediated transcription factors SMAD2/3 are detectable in nuclei of primordial GCs, suggesting that this pathway is active in these cells^6^. It is well established that TGFβ signalling is important for maintaining growth arrest in a range of cell types by directly inhibiting *Myc*^7–9^, as well as regulating the expression of other cell cycle proteins^10,11^. Cell proliferation initially involves cell cycle progression from G_0_-G_1_^12^. Typically, this occurs through growth factor stimulated expression of cyclin D, which then binds to cyclin-dependent kinase (CDK) proteins, precipitating release of E2F transcription factors to drive cell cycle progression^12–14^. In many cell types, cyclin D-CDK complexes can also be inhibited by P27, creating a stable trimeric complex that maintains cell cycle arrest^15^. In the ovary, in contrast to other D-type cyclins, cyclin D2 (CCND2) mRNA is detectable in GCs of follicles across a range of stages and knockout of both cyclin D2 (*Ccnd2*^*−/−*^) and *p27*^*−/−*^ exhibit dis-regulated early follicle development with major effects on fertility^16–18^.

We therefore carried out detailed analysis of CCND2 and P27 in the context of arrested primordial and early developing follicles, focussing on the relationship of these factors with TGFβ signalling in GCs. Using the C57/Bl6 mouse as a model, we show that SMAD3 specifically localises to GCs of small follicles where it directly promotes the expression of *Ccnd2* and represses *Myc*. The CCND2 protein is detectable in complex with the inhibitory factor P27 and together highlights a mechanism that potentially maintains primordial GC arrest, whilst ensuring they are poised ready to proliferate. Interestingly, the loss of SMAD3 in GCs of growing follicles is associated with elevated mTOR signalling, potentially explaining a dual mechanism of TGFβ signalling in follicle growth and arrest.

## Results and Discussion

### SMAD3 is predominantly expressed in GCs of primordial follicles

**Figure 1:**
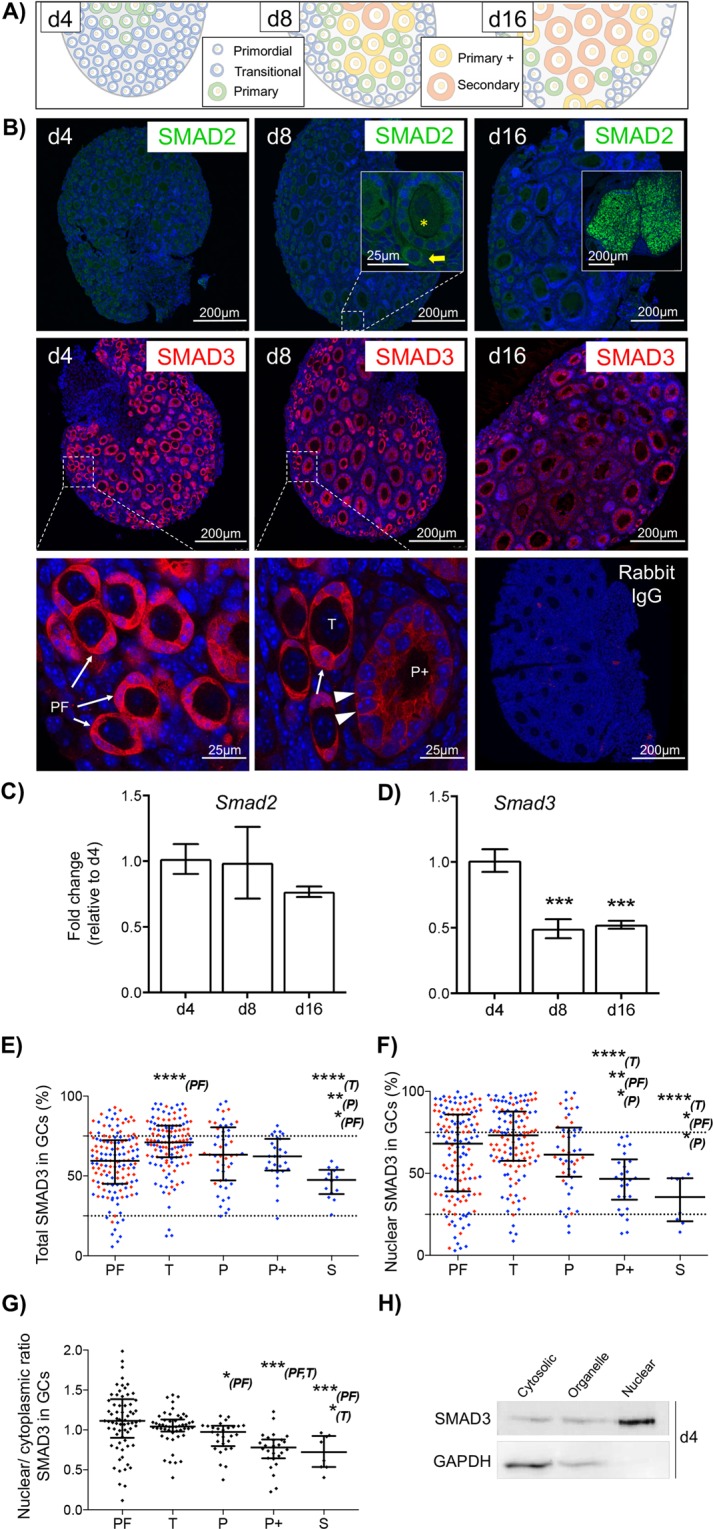
SMAD3 is predominantly expressed in GCs of primordial follicles. (**A**) Diagram showing the follicle composition of the prepubertal mouse ovary at 4, 8 and 12 days of age (d4, d8, d12). (**B**) Immunofluorescent localisation of SMAD2 and SMAD3 protein in the prepubertal mouse ovary. SMAD2 (green) was weak/undetectable in prepubertal ovaries, including primordial (yellow arrow) and early growing primary follicles (yellow asterisk), but was strong in corporal lutea from adult ovary (d16 inset). SMAD3 (red) localised to the nuclei and cytoplasm of GCs of primordial and transitional follicles (arrows), with nuclear expression appearing weaker in GCs of growing primary-plus follicles (arrowheads). Cell nuclei are counterstained with DAPI (blue). Rabbit IgG is shown as a negative control. (**C,D**) Relative expression of *Smad2* and *Smad3* mRNA by qPCR in d4, d8 and d16 ovaries. (**E–G**) Quantitative analyses of SMAD3 immunofluorescence. (**E**) Data represents total SMAD3 staining intensity in GCs by follicle stage. Each point represents the % positive pixels in the GC compartment for an individual follicle. (**F**) Nuclear SMAD3 intensity in GCs by follicle stage. Each point represents the % positive pixels in individual GC nuclei within each follicle stage. Points in red and blue represent measurements from d4 and d8 ovaries, respectively. (**G**) Nuclear/cytoplasmic ratio of SMAD3 in GCs by follicle stage. Each point represents the % positive pixels in all GC nuclei relative to % positive pixels in the GC compartment for an individual follicle obtained from d8 ovaries. Dashed lines (**E,F**) demarcate lower and upper quartiles to represent proportions of samples that were considered low, medium and high intensity staining, respectively. (**H**) Subcellular expression of SMAD3 by western blotting in protein lysates from d4 mouse ovaries. Samples were separated into cytosolic, membrane-bound organelle, and nuclear fractions. The cytoplasmic protein GAPDH is included as a control. Gel images have been cropped from originals provided in Fig. S1. PF, primordial; T, transitional; P, primary; P + , primary plus, S, secondary follicle. Data in C, D are means ± SEM (n = 5 ovaries/age) and differences are relative to d4. Data in E, F, G are medians ± interquartile ranges and differences are relative to the follicle stage in parentheses. *P < 0.05, **P < 0.01, ***P < 0.001, ****P < 0.0001.

Both SMAD2 and SMAD3 mediate TGFβ signalling and regulate gene expression^10,11,19^; however, whether both or either SMAD is important in this context is not known. In this study, we have used ovaries from C57/Bl6 mice at postnatal day 4, 8 and 16 as a natural developmental paradigm to analyse gene expression in primordial, early activated (transitional), and growing follicles (primary, primary+ , secondary), respectively^20,21^ **(**Fig. 1A**)**. Immunofluorescent localisation of SMAD2 was very weak in ovaries at each age, while SMAD3 was specifically expressed in GCs of small follicles, notably in the earliest primordial stages as exemplified in sections from day 4 ovaries **(**Fig. 1B**)**. Consistent with this, the level of *Smad2* mRNA, although detectable, did not vary between the different ovary ages **(**Fig. 1C**)**, whereas *Smad3* mRNA expression was reduced in day 8 and 16 ovaries in which the relative proportion of primordial follicles is reduced (Fig. 1D). Based on these findings we focused on SMAD3 for further analyses.

When SMAD3 immunofluorescence intensity values were plotted by follicle stage, a bi-phasic pattern was evident, with total GC-expression being highest in transitional follicles that are poised to initiate growth, and lowest in more advanced secondary-staged growing follicles (Fig. 1E). Since SMAD3 is a cytoplasmic signalling mediator and nuclear transcription factor^19,22^, we analysed GC expression in both cellular compartments. In GC nuclei SMAD3 was highly variable; however, the median expression was similar between primordial, transitional and primary staged follicles, and was lower from the primary plus stage (Fig. 1F). Nuclear SMAD3 in each follicle was then normalised against cytoplasmic expression to determine if the ratio was equal throughout follicle development. The results, however, show that SMAD3 was similarly located in the nuclei and cytoplasm of GCs in primordial and transitional follicles, with a relative reduction in nuclear expression from the primary stage, indicating that nuclear exclusion occurs prior to the general loss of SMAD3 expression in GCs (Fig. 1G). Sub-cellular fractionation of protein lysates from day 4 ovaries further confirmed that SMAD3 was detectable in both nuclear and non-nuclear fractions (Fig. 1H). Since SMAD3 exhibits a DNA binding domain^22^, our findings support a role for SMAD3 as a potential transcriptional regulator in GCs of primordial follicles.

### CCND2 and P27 are maintained in GCs of primordial follicles but decrease as follicles initiate growth

**Figure 2:**
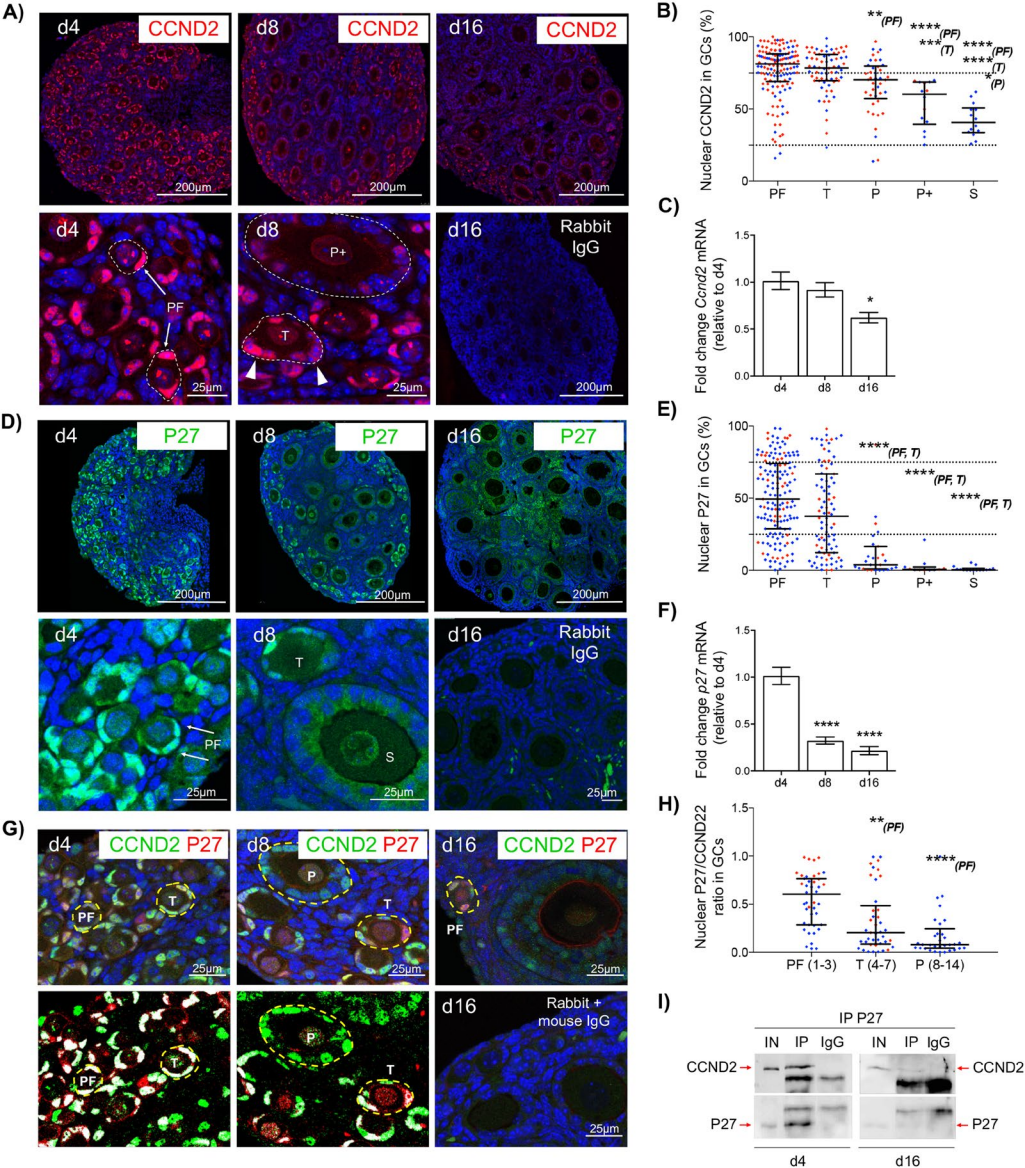
CCND2 and P27 are maintained in GCs of primordial follicles but decrease as follicles initiate growth. (**A**) Immunofluorescent localisation of CCND2 (red) in the prepubertal mouse ovary. High power images show strong (persistent) nuclear CCND2 staining in GCs of primordial follicles (PF; arrows). Transitional follicles (T) show nuclei with variable CCND2 (arrow heads) whereas larger primary plus staged follicles (P+) show weak CCND2 expression. (**B**) Nuclear CCND2 protein expression in GCs by follicle stage. Each point represents the % positive pixels for all GC nuclei in an individual follicle. **(C)** Relative expression of *Ccnd2* mRNA by qPCR in d4, d8 and d16 ovaries. **(D)** Immunofluorescent localisation of P27 (green) in the prepubertal mouse ovary. High power images (lower panels) show strong nuclear P27 staining in GCs of primordial follicles (PF; arrows). Transitional follicles (T) show nuclei with variable P27 whereas larger secondary staged follicles (S) show weak P27 expression. **(E)** Nuclear P27 protein expression in GCs by follicle stage. Each point represents the % positive pixels for all GC nuclei in an individual follicle. **(F)** Relative expression of *p27* mRNA by qPCR in d4, d8 and d16 ovaries. **(G)** Co-localisation of CCND2 and P27 in the prepubertal mouse ovary. After image processing (d4 and d8; lower panels), only positive pixels in the green and red channels are shown, highlighting co-localisation in white. A negative IgG control (d16; lower right) is shown. **(H)** P27/CCND2 ratio in individual GCs by follicle stage. Each point represents the % positive pixels for P27 relative to % positive pixels for CCND2 an individual nucleus. Numbers in parentheses refer to the GC number range for each follicle stage. **(I)** Western blot of P27 and CCND2 following co-immunoprecipitation from d4 and d16 ovaries. Protein samples (12.5 µg) were immunoprecipitated using anti- P27 (IP) or non-specific rabbit IgG (IgG; control). Non-immunoprecipitated protein (5 µg) was used as positive control (IN). Gel images have been cropped from originals provided in Fig. S2. PF, primordial; T, transitional; P, primary; P+, primary plus, S, secondary follicle. Nuclei in A, D, G are labelled with DAPI (blue). Data in B, E, H are medians ± interquartile ranges and differences are relative to the follicle stage in parentheses. Points in red and blue represent measurements from d4 and d8 ovaries, respectively. Data in C, F are means ± SEM (n = 5 ovaries/age) and differences are relative to d4. *P < 0.05, **P < 0.01, ***P < 0.001, ****P < 0.0001.

The expression of D-type cyclins occurs early in the cell cycle^12,15^; therefore, the localisation and regulation of CCND2, as well as the inhibitory partner P27 in quiescent primordial follicles were investigated. CCND2 was detectable in GC and oocyte nuclei, with relatively strong staining evident in GCs of small, single layered follicles (Fig. 2A). Strong expression (intensity values >75%) of CCND2 was detectable in GCs of over 50% of primordial follicles analysed, with a relative reduction in expression from the primary stage onwards (Fig. 2B). Consistent with this, *Ccnd2* mRNA levels in whole ovaries were significantly reduced in older ovaries containing proportionately more growing follicles, relative to day 4 ovaries (Fig. 2C). As with CCND2, P27 protein localised to GC nuclei of small follicles (Fig. 2D). Staining was highly variable between primordial follicles; however, the nuclear expression of P27 significantly declined in the majority of growing follicles from the primary stage onwards (Fig. 2E). This loss of expression in developing follicles reflected *p27* mRNA expression in whole ovaries, which was significantly reduced in day 8 and 16 ovaries, relative to day 4 (Fig. 2F).

Both proteins co-localised in GC nuclei of small follicles regardless of age (Fig. 2G). We then focussed on d4 and d8 ovaries due to the preponderance of primordial and small activated follicles. Although the degree of co-localisation in each cell varied widely within follicles and between follicle stages, the ratio of P27 to CCND2 declined from the transitional stage onwards. Thus, as follicles activate, P27 is initially reduced while CCND2 persists (Fig. 2H). In support of this, both CCND2 and P27 co-immunoprecipitated in protein lysates from day 4 ovaries. However, in day 16 ovaries, although CCND2 was detectable by western blot, the same protein was beyond the limits of detection by western blotting following immunoprecipitation with P27 (Fig. 2I). Together these data indicate that CCND2 and P27 exist in molecular complexes in GCs of primordial follicles. Consistent with a role for P27 as a cell-cycle inhibitory factor^15^, it is likely that P27 is inhibiting the activity of CCND2 in primordial GCs, thereby participating in maintaining the quiescent phenotype of primordial follicles.

### SMAD3 directly regulates *Ccnd2* and *Myc* expression in primordial follicles

**Figure 3:**
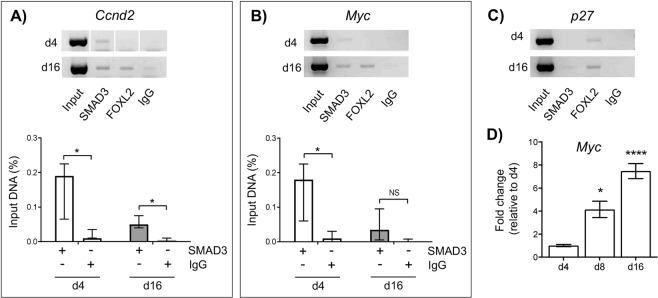
SMAD3 directly regulates *Ccnd2* and *Myc* expression in primordial follicles. (**A–C)** Amplification of target binding sites upstream of the *Ccnd2, Myc* and *p27* genes following ChIP. Representative gel images showing amplification of DNA after immuno-precipitation with anti-SMAD3, anti-FOXL2 (GC expressed transcription factor – control), or non-specific antibodies (IgG) in d4 and d16 mouse ovaries. A positive sample of chromatin (Input) prior to immunoprecipitation is also shown (upper panels). Relative quantification of anti-SMAD3 IgG binding to *Ccnd2* (lower panel A) and *Myc* (lower panel B) gene promoters in d4 and d16 ovaries by qPCR. % input was calculated by normalizing anti-SMAD3 IgG and non-specific IgG (control) against input DNA as described in the *materials and methods*. Each group (d4, d16) contained 5 repeats (n = 5 ovaries). Data are medians ± interquartile range and statistical differences are relative to IgG control. *P < 0.05, NS: Not significant. Note the *Ccnd2* gel is a composite as indicated with divisional space and all gel images have been cropped from originals provided in Fig. S3. **(D)** Relative expression of *Myc* mRNA by qPCR in d4, d8 and d16 ovaries. Means ± SEM (n = 5 ovaries/age) and differences are relative to d4. *P < 0.05, ****P < 0.0001.

Since the transcription factor SMAD3 is expressed in GC nuclei of primordial/transitional follicles alongside CCND2 and P27, we then investigated whether SMAD3 has the ability to directly bind and regulate the expression of *Ccnd2* and *p27* in this context. The immediate early gene *Myc* was also used as a positive control, since it is a well-known target of SMAD3 and is involved in cell-cycle progression^7–9^. In day 4 and day 16 ovaries, SMAD3 was detected on the promoter of *Ccnd2* using ChIP-PCR, which was significant when quantified in relation to non-immune control IgG **(**Fig. 3A**)**. This is consistent with the observed reduction in *Ccnd2* gene expression in ovaries of *Smad3*^*−/−*^ mice^23,24^. SMAD3 was also detectable on the promoter of the *Myc* gene in day 4 ovary samples; however, this binding was not significantly different from control IgG in day 16 ovary samples **(**Fig. 3B**)**. By comparison, SMAD3 binding to the *p27* promoter was undetectable by ChIP-PCR in day 4 ovaries, and barely detectable in day 16 ovaries **(**Fig. 3C**)**. Therefore, it is unlikely that *p27* is a direct target of SMAD3 in small follicles.

Since *Myc* has a well-ascribed role in promoting cell proliferation^25,26^, we then quantified expression in immature ovaries. The increase in *Myc* mRNA expression in day 8 and day 16 ovaries relative to day 4 ovaries (Fig. 3D) suggests that SMAD3 acts to repress *Myc* transcription in primordial follicles. Conversely, the decrease in *Ccnd2* mRNA expression in day 16 ovaries relative to day 4 ovaries observed previously (Fig. 2A–C) shows that SMAD3 likely promotes cyclin D2 expression in primordial follicles. These findings imply that TGFβ-SMAD3 signalling acts directly on GCs of primordial follicles to differentially regulate genes involved in cell cycle regulation.

### Short term exposure to TGFβ1 promotes mTOR signalling in neonatal mouse ovaries

**Figure 4:**
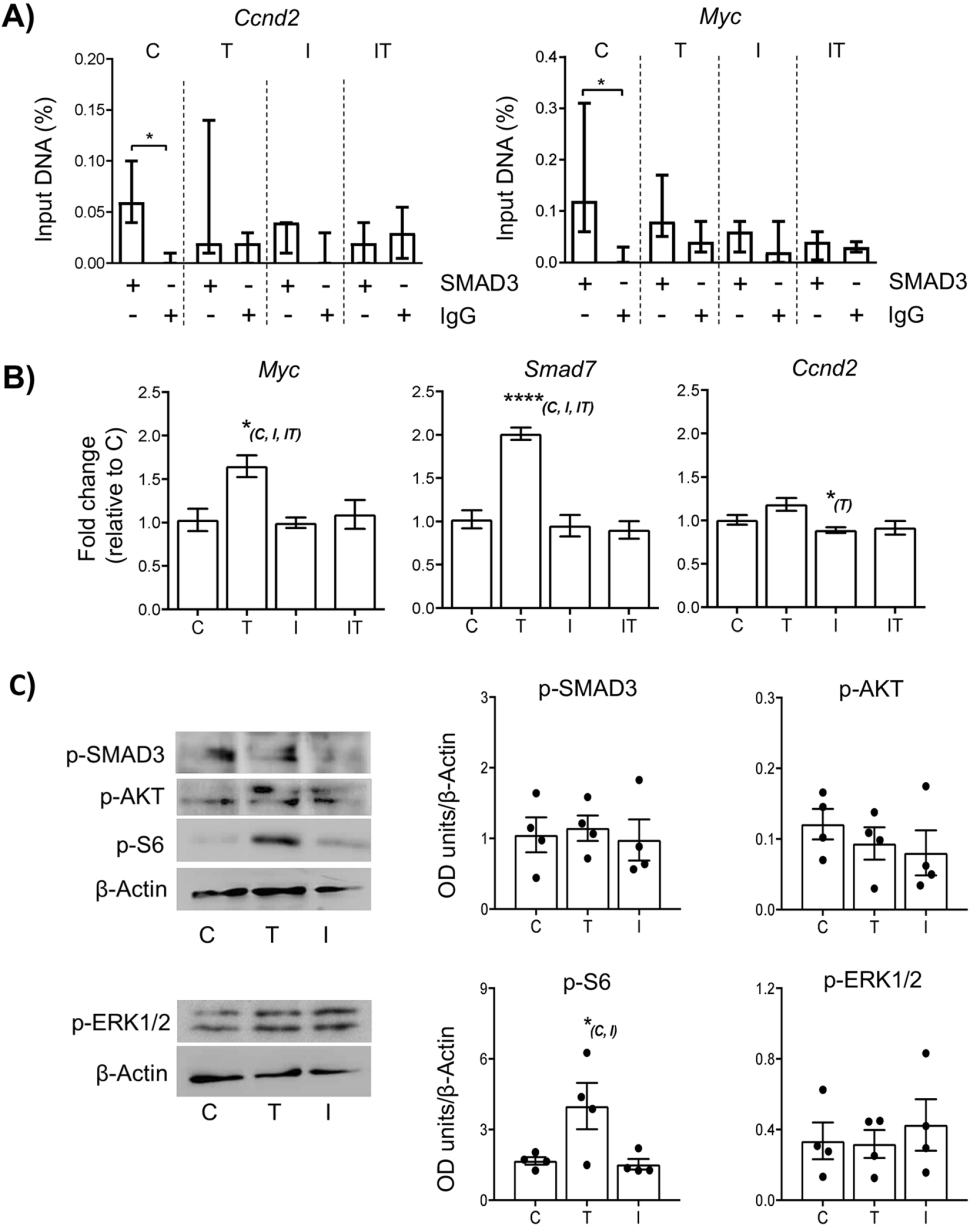
Short term exposure to TGFβ1 promotes mTOR signalling in neonatal mouse ovaries. Whole neonatal mouse ovaries (d4) were maintained *in vitro* and exposed for 2 hours in either 1 µM DMSO (control; C), 10 ng/ml of TGFβ1 ligand (T), or 1 µM A83-01 inhibitor (I). An additional group was exposed to 1 µM A83-01 inhibitor followed by 10 ng/ml of TGFβ1 ligand (IT). Ovaries were analysed for gene and protein analysis immediately following treatment. **(A)** Relative quantification of SMAD3 binding to *Ccnd2* and *Myc* gene promoters in cultured ovaries by ChIP-qPCR. Each group contained 5 ovaries (n = 5). Data are medians ± interquartile range and statistical differences are relative to IgG control (*P < 0.05). (**B**) Relative expression of *Myc, Smad7* and *Ccnd2* mRNA in cultured ovaries. Each group contained 5 ovaries (n = 5). Data are means ± SEM; *P < 0.05 vs group indicated in parentheses. **(C)** Expression of phospho-intermediates in the canonical TGFβ (p-SMAD3), PI3 kinase/Akt (p-AKT), p44/42 MAPK (p-ERK1/2) and mTOR (p-S6) pathways in cultured ovaries by western blot. Example blots are shown (left-hand panels; originals provided in Fig. S4) and levels quantified by normalization against beta-actin (loading control). Data represents four samples from each group, each from an independent blot. *P < 0.05 vs group indicated in parentheses.

Considering the above observations, we then investigated the effect of an altered TGFβ environment on small follicle development *in vitro*. Previous studies have shown that long-term culture of neonatal rodent ovaries ( > 7 days) with a TGFβ ligand can influence primordial follicle viability^27–29^. In cell culture models, changes in SMAD3-regulated gene expression have been observed as little as 30 minutes after stimulation with TGFβ ligands^30,31^. Furthermore, a 60–90 min exposure of kit ligand or the pathway inhibitor imatinib to post-natal day 7 mouse ovaries in culture was sufficient to cause a significant shift in FOXO3a translocation in oocytes^32^. We therefore evaluated effects on gene expression and signalling following a brief 2-hour exposure to TGFβ1 ligand and A83-01, a Type I receptor inhibitor, in day 4 ovaries *in vitro*. Consistent with *in vivo* findings, SMAD3 bound to the promoter of *Ccnd2* and *Myc* in unstimulated (control) ovaries. By comparison, binding to *Ccnd2* and *Myc* was no longer evident in ovaries exposed to either TGFβ1, A83-01, or a combination of TGFβ1 and A83-01 (Fig. 4A), indicating that modulation of TGFβ signalling causes dissociation of SMAD3 from the promoters of these genes. Moreover, dissociation of SMAD3 to target gene promoters was not able to be over-ridden by exposure to TGFβ1. Interestingly, this change in binding was associated with elevated expression of the pro-proliferation factor *Myc* in ovaries exposed to TGFβ1. Myc is an early response gene and in other studies *Myc* mRNA reaches maximum levels after 2–3 hours after growth factor stimulation, whereas induction of *Ccnd2* mRNA peaks around 4 hours^33,34^. The latter point may explain why the loss of SMAD3 occupancy on the *Ccnd2* promoter did not effect *Ccnd2* mRNA expression in the same way as *Myc* (Fig. 4B). Elevated transcript levels of *Smad7* – a negative regulator of TGFβ signalling^35,36^ were also observed, suggesting one effect of increased exposure to TGFβ1 is to inhibit the action of SMAD3 **(**Fig. 4B**)**. In ovaries exposed to A83-01 or a combination of TGFβ1 and A83-01, Myc expression was unchanged. As Myc is normally repressed in cells with low turnover, it is possible that other transcriptional repressors independent of TGFβ signalling, such as WT1^37,38^, are active in primordial GCs, although further studies would be required to confirm this.

Since exposure to A83-01 and TGFβ1 led to a similar loss of SMAD3 promoter binding, but with differential effects on *Myc* and *Smad7* expression, we sought to determine if this was associated with elevated SMAD3 signalling, or activation of SMAD-independent pathways. Levels of p-SMAD3, p-AKT and p-ERK1/2 proteins did not vary between TGFβ1, A83-01 or control groups. By comparison, the mTOR mediator p-S6 was significantly increased in ovaries exposed to TGFβ1 in comparison to ovaries exposed to A83-01 or control (Fig. 4C), indicating that elevated TGFβ signalling in neonatal ovaries leads to early effects on the mTOR pathway.

### Acute modulation of TGFβ signalling causes stage-specific effects on follicle development

**Figure 5:**
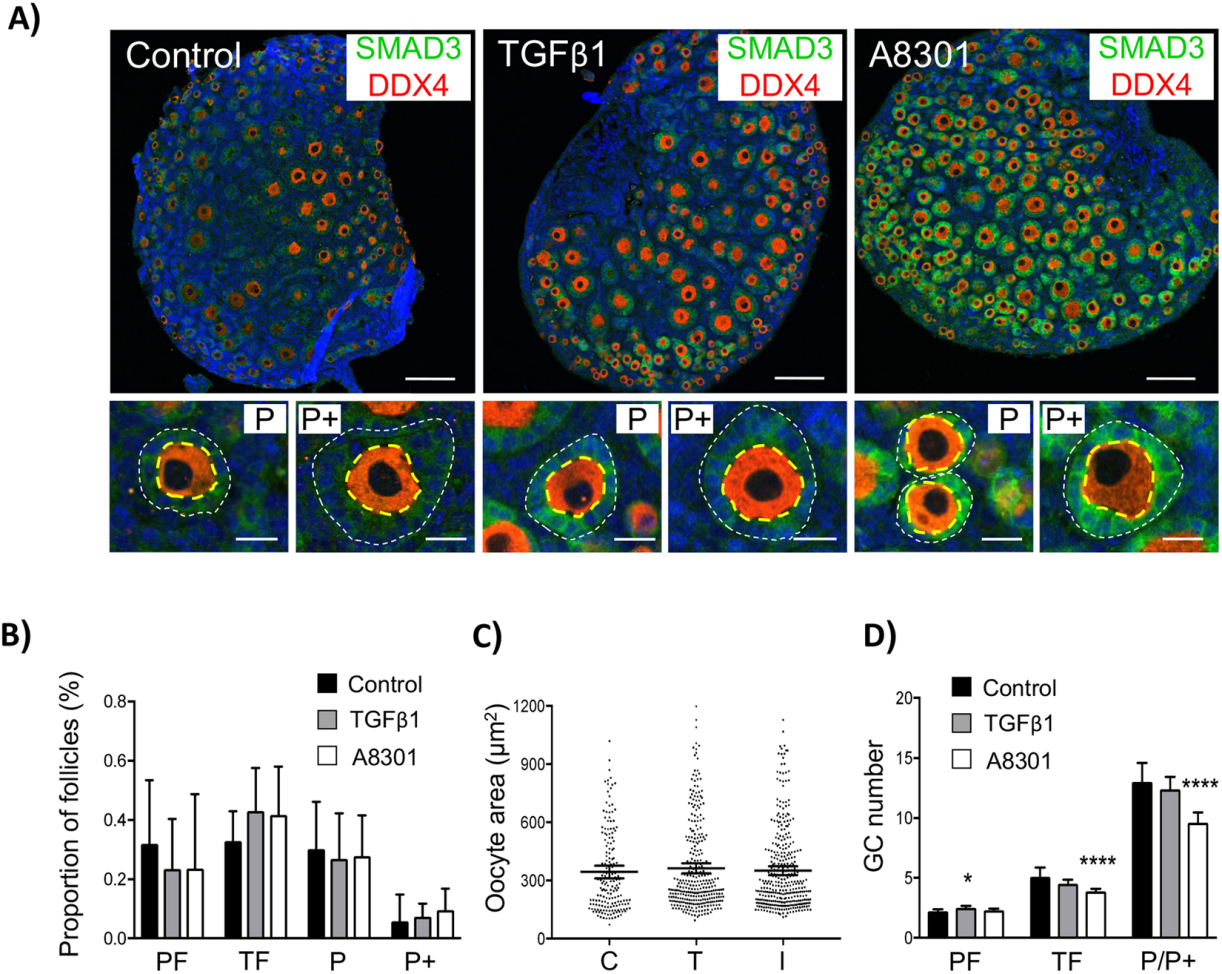
Acute modulation of TGFβ signalling causes stage-specific effects on follicle development. Whole neonatal mouse ovaries (d4) were maintained *in vitro* and exposed for 2 hours with either 10 ng/ml of TGFβ1 ligand (T), 1 µM A83-01 inhibitor (I), or 1 µM DMSO (C). Ovaries were then placed in basic culture media for two additional days prior to morphological analysis. **(A)** Confocal images of sections of cultured ovaries immunolabelled with DDX4 (red) and SMAD3 (green) to highlight oocytes and GCs, respectively. Cell nuclei are counterstained with DAPI (blue). Low power images (upper panels) showing general distribution of follicles in each group. Scale bars = 100 µm. High power images (lower panels) showing examples of early growing primary (P) and primary plus (P+) follicles in each group as classified by oocyte size. Note the smaller size of the GC-layer in follicles exposed to the inhibitor. Scale bars = 20 µm. **(B)** Proportion of follicles classified by follicle stage. **(C)** Distribution of oocyte size by treatment. **(D)** Mean GC number per follicle. Quantitative data from cultured ovaries (**B–D**) was obtained from six different ovary sections per treatment group (n = 6 ovaries/group). Classification was based on oocyte size (Fig. S5). Data show means ± 95% CI. *P < 0.05 and ****P < 0.0001 vs control. PF, primordial; T, transitional; P, primary; P+, primary plus follicle.

Finally, in order to evaluate the consequence of an acutely altered TGFβ environment, ovaries were returned to control media for an additional two days. Processed ovaries were then stained with SMAD3 and DDX4 as markers of the GC compartment and oocytes, respectively, for morphological assessment and quantification. The overall distribution of follicles was similar between groups, although GCs of growing primary follicles in ovaries exposed to A83-01 had failed to adequately cuboidalise (Fig. 5A). When follicles were classified according to oocyte size, TGFβ1 or A83-01 had no effect on stage of follicle development (Fig. 5B). Likewise, exposure to TGFβ1 or A83-01 had no effect on oocyte size (Fig. 5C). Interestingly, brief exposure to TGFβ1 ligand promoted a small, but measurable increase in the mean GC number of follicles classified as primordial, suggesting that 2 hours of elevated TGFβ1 was sufficient to promote GC proliferation but not sufficient enough to promote significant oocyte growth. By comparison, exposure to A83-01 had no effect on GCs of primordial follicles but caused a significant reduction in mean GC number in follicles classified as growing, indicating that inhibition of TGFβ receptors inhibits normal GC proliferation in activated follicles (Fig. 5D). It should also be noted that very few apoptotic cells were detectable in any of the groups as determined by TUNEL labelling **(**Fig. S6**)**. The reduction in GC number along with inadequate cuboidalisation indicates that inhibition of TGFβ receptors likely impairs normal GC proliferation in activated follicles. Thus, although SMAD3 is reduced in GCs of growing follicles (Fig. 1), TGFβ signalling is still essential, which is consistent with the well-ascribed role of other TGFβ ligands that signal through the same Type I receptors, such as GDF9^39–42^ and activin^43–46^ in supporting early follicle development.

## Conclusions

The ovarian reserve consists of a limited pool of primordial follicles held in a state of developmental arrest^1,3–5^. The molecular programme that maintains this arrest, and conversely regulates activation and growth is largely unknown. We recently presented a detailed analysis highlighting an inverse relationship between nuclear SMAD2/3 expression and cell proliferation in GCs of mouse ovaries, suggesting a role for TGFβ signalling in primordial follicle maintenance and activation^6^. Here, we show that SMAD3 recapitulates the pattern of SMAD2/3 and is associated with expression of the cell cycle regulators cyclin D2 and P27. The specific localisation of these proteins was similar in ovaries derived from d4 and d8 mice, each with different populations of developing follicles, further highlighting a stage-specific, rather than age-dependent role in in GCs of non-growing and early growing follicles (Fig. S7). In non-growing follicles, it is unlikely that SMAD3 directly regulates P27 in this context; instead, SMAD3 binds directly to *Ccnd2* and *Myc* to differentially regulate these genes, which may be important for ensuring GCs are ‘poised’ in a state ready to progress through the cell cycle. The presence and accumulation of CCND2 in complex with P27 suggests a mechanism for primordial follicle maintenance, where P27 may prevent GC cycle progression by inhibiting cyclin-CDK activity^15^. This hypothesis is consistent with other studies reporting an inhibitory effect of P27 on early follicle activation^16,47^. Although the mechanism leading to a reduction in P27 activity in this context is currently unknown, it is clear that follicle growth is also associated with a reduction in accumulated CCND2 and SMAD3 – the latter also allowing de-repression of *Myc*. CCND2 and SMAD3 are important regulators of the cell cycle and although their steady-state expression is reduced in growing follicles, both proteins are still expressed at a moderate level in GCs **(**Figs 1 and 2**)**. Furthermore, briefly exposing ovaries to an elevated environment of TGFβ led to a slight increase in GC proliferation. This occurred following an immediate uncoupling of SMAD3 from *Ccnd2* and *Myc* gene promoters alongside elevated mTOR signalling. Activation of mTOR signalling in primordial GCs is a key early event in follicle initiation^48^, has been implicated in TGFβ-driven models of epithelilal-mesenchymal transition^49^, and plays an important role more generally in cellular growth and proliferation^50^.

**Figure 6:**
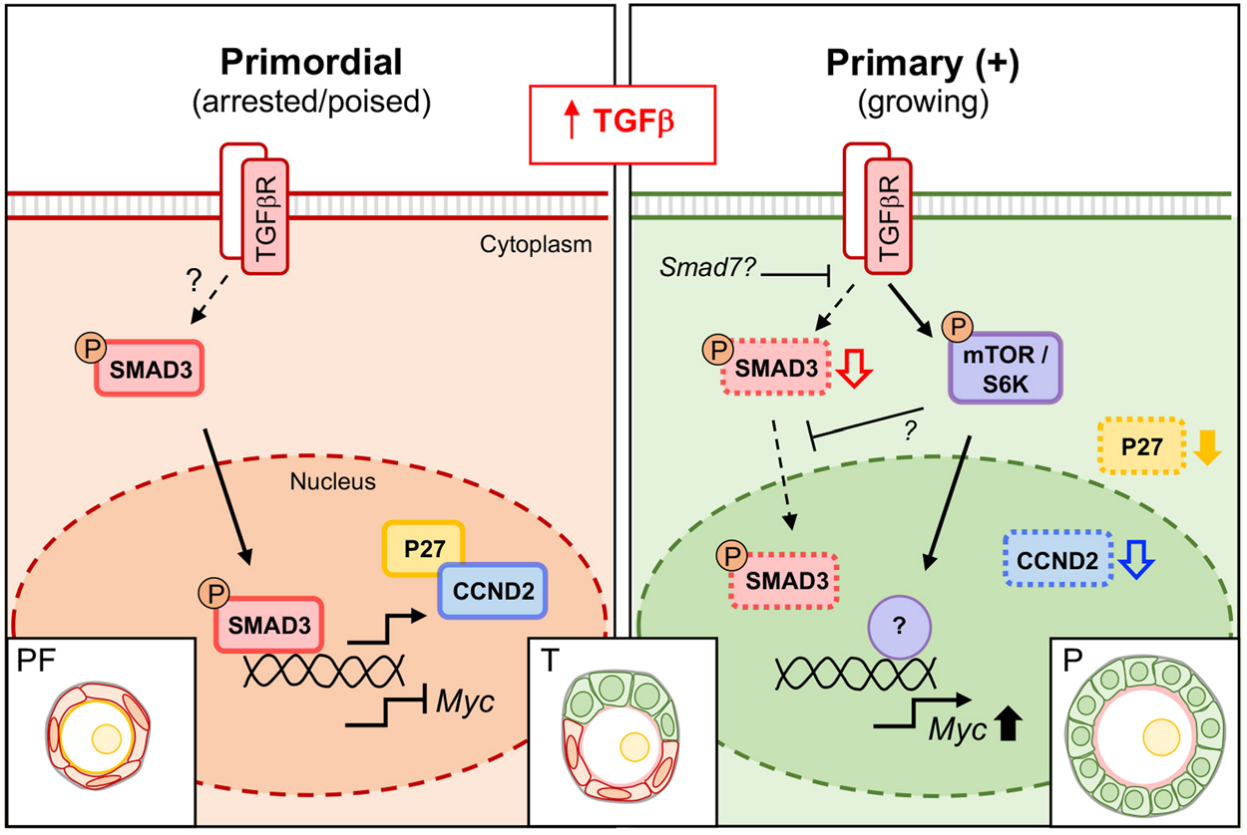
Model of cell cycle regulation in GCs of small follicles. Arrested GCs of primordial follicles express the transcription factor SMAD3, which directly promotes expression of CCND2 and represses *Myc*. In this ‘poised’ state, CCND2 is bound by the inhibitory factor P27. In transitional GCs, P27 dissociates from CCND2 and SMAD3 expression transiently increases, possibly via elevated TGFβ signalling, allowing maintenance of CCND2 expression for cell cycle progression. Increased SMAD3 promotes expression of factors such as *Smad7*, which act to inhibit SMAD3 in a self-regulatory manner. The eventual loss of SMAD3 leads to de-repression of *Myc* and activation of SMAD-independent TGFβ signalling involving mTOR to drive GC proliferation in growing follicles. Solid black line arrows indicate predominant signalling pathway. Coloured block arrows indicate moderate (unfilled arrows) or substantial (filled arrows) changes in expression relative to primordial stage. Note in growing follicles, although SMAD3 and CCND2 are reduced, they are still detectable and play an essential role in regulation of the GC cycle. PF, primordial; T, transitional; P, primary follicle.

Based on these findings, we propose a multi-step mechanism of follicle activation that initially involves a basal level of SMAD3 expression and nuclear activity to prepare GCs for proliferation. It remains to be determined whether this activity in primordial GCs is dependent on receptor-mediated signalling, and if so, which is the principle ligand involved; however, increased TGFβ1 ligand exposure promotes a shift to a SMAD-independent pathway, which drives cell proliferation and eventual follicle activation **(**Fig. 6**)**. The role of other TGFβ superfamily members and the cross-talk involved in regulating SMAD signalling in this context will also be important to establish. Although this study has used the mouse as a model organism, revelation of the molecular framework that underscores the phenotype of pre-granulosa cells in other species is essential for understanding how the ovarian reserve is maintained throughout the reproductive lifetime of the female.

## Materials and Methods

### Animals and tissue collection

All ovary tissues used in this study were obtained from wild-type C57Bl6 mice housed in the Biological Services Unit at the University of Sheffield in compliance with the Animals (Scientific Procedures) Act, 1986. Mice were killed by a registered practitioner (Schedule 1 procedures) with approval from the University of Sheffield Training and Competency Officer. For total RNA, protein extraction and ChIP experiments, ovaries from immature mice (day 4, 8 and 16) were dissected and either placed in culture (day 4 only) or snap frozen in liquid nitrogen and stored at −80 °C. For immunostaining, ovaries were immersed in 10% neutral buffered formalin solution (Sigma-Aldrich) for 24 hours and processed in paraffin blocks and sectioned at 5 μm.

### Neonatal mouse ovary culture

Ovaries were placed in 24 mm Transwell plates containing membrane inserts (Corning; Sigma). Culture medium consisted of Waymouth medium 752/1 (Life Technologies) supplemented with 10% fetal bovine serum (FBS; Sigma), 0.23 mM pyruvic acid (Sigma), 10 µg/ml streptomycin sulfate (Sigma), 75 µg/ml penicillin G (PENK; Sigma) and 0.3 mg/mL BSA. Ovaries were maintained in a 37 °C incubator (5% CO_2_) for 24 h prior to any treatment. For the assessment of the molecular changes under different TGFβ conditions (SMAD3 binding and gene regulation) ovaries were exposed for 2 h in serum free culture media, supplemented with either TGFβ1 ligand (10 ng/ml; R&D systems, 240-B-002), A83-01 (1 µM; MedChem Press, 2939), a selective inhibitor of Type 1 TGFβ receptors ALK4, ALK5 and ALK7^51^, a combination of TGFβ1 (10 ng/ml) and A83-01 (1 µM), or DMSO (control; same volume as for A83-01; Sigma). A single time point (2 hours) and concentration for TGFβ1 ligand (10 ng/ml) and A 83-01 inhibitor (1 µM) were selected based on previous studies^27–29,51,52^. Immediately following treatment, ovaries were collected and either fixed and processed for ChIP-qPCR or flash frozen in liquid nitrogen for gene expression analysis. For the assessment of the morphological changes, ovaries were exposed for 2 h in serum free culture media followed by supplementation as above. In the TGFβ1 treatment group, the addition of A83-01 inhibitor for an extra 1 hour was used to block further TGFβ1 signalling as previously described^53^. Treatments were then washed out and ovaries were left in culture for two days before processing.

### Immunofluorescence staining

Sections were dewaxed before microwaving in 0.1 M citrate buffer (pH6.0). Sections were blocked with CAS universal blocking reagent (ThermoFisher) for 20 minutes at room temperature to reduce non-specific binding. Specific antibodies against SMAD2 (0.59 µg/ml, #5339, Cell Signaling Technology, MA, USA), SMAD3 (0.75 µg/ml, #9523, Cell Signaling), SMAD2/3 (0.25 µg/ml, 133098, Santa Cruz Biotechnology, TX, USA), CCND2 (0.5 µg/ml, sc-593, Santa Cruz Biotechnology, TX, USA), DDX4 (2.2 µg/ml, ab13840, Abcam, Cambridge, UK) and P27 (1 µg/ml, sc-528, Santa Cruz or 0.5 µg/ml, sc-1641 for co-localisation) were diluted in blocking solution and incubated overnight at 4 °C. A species-specific isotype control IgG antibody at the same concentration (normal rabbit IgG I-1000 and normal mouse IgG I-200, Vector Laboratories) were included in each experiment. Sections were washed in PBS and incubated with secondary antibodies (either donkey anti-rabbit AlexaFluor® 488 or AlexaFluor® 555; donkey anti-mouse AlexaFluor® 488 and AlexaFluor® 555, each 1:400; Invitrogen). Sections were mounted with a drop of Prolong® Gold anti-fade reagent with DAPI (ThermoFisher) and were imaged using an inverted Leica SP5 confocal laser-scanning microscope (Leica Microsystems, Wetzlar, Germany).

### Image analysis and quantification of imunofluorescence

Quantification of GC factors *in situ* was performed from a total of 7 ovaries (four day 4 and three day 8 ovaries) using the largest cross section per ovary. Confocal images were stitched together using DoubleTake (http://echoone.com/doubletake/) to create a high-resolution composite of each section. Images were obtained as an 8-bit RGB stack file and the threshold for each channel in every composite section was set up with the same criteria across the different sections of the same staining. Pixels above the threshold were considered as ‘positive pixels” while the remaining pixels were registered as negative. The oocyte cytoplasm was used as a baseline setting (due to lack of specific staining) and threshold settings were normalised against this to account for variation between slides. Follicles with a visible oocyte nucleus were selected with the region of interest (ROI) manager tool of ImageJ software (https://imagej.nih.gov/ij/). In order to quantify each of the candidate proteins in different ROIs, the area of positive pixels “Area fraction” was calculated and referred as “Intensity” for simplicity. ROIs from each follicle were selected manually using the DAPI channel and the “measure and label” tool, which allowed the intensity of each ROI in either the green or red channel to be quantified. Follicles were classified by the number of GCs and oocyte area **(**Fig. S5**)**. For each follicle, measurements were recorded for the intensities of individual GC nuclei. For SMAD3 analysis, the intensity of the entire GC compartment was also measured and the ratio between the geometric mean of the intensities for the GC nuclei, and the geometric mean for the entire GC compartment were calculated and plotted as a measure of nuclear:cytoplasm expression. CCND2 and P27 co-localisation was analysed with the “Colocalization threshold” plugin from ImageJ. A minimum of 20 individual GC nuclei from primordial and transitional follicles were selected from a single day 4 and day 8 ovary section. The ratio of intensities between CCND2 and p27 was represented as (P27/CCND2). For cultured ovaries, sections were stained with antibodies against DDX4 and SMAD3 and image analysis was performed as described above. When more than one section from the same ovary was used, a minimum separation of 2 sections (10 µm) from the largest cross-section was considered to avoid double counting.

### Gene expression analysis

Total RNA was isolated from ovaries using RNeasy® Micro Kits (QIAGEN, Crawley, UK). Concentration and purity of all RNA samples was assessed using the Agilent 2100 Bioanalyzer. Samples with an RNA integrity number (RIN) between 8 and 10 were used for gene expression analysis. Fifty ng of RNA from each sample was converted to cDNA using the SuperScript III Reverse Transcription kit (Invitrogen; ThermoFisher). Quantivative PCR (qPCR) using 500 nM of primers for *Smad2*, *Smad3*, *Smad7*, *Myc, Ccnd2* and *p27* **(**Table S1**)** were used with the SensiFAST™ SYBR® Hi-ROX kit (Bioline). Efficiencies were 90–130% for all primers. Samples (1 µl per reaction) and the same volume of distilled water (negative control) were run in duplicate in 384 well-plates. qPCR conditions were as follows: activation at 95 °C for 2 minutes and then 40 cycles of amplification (3 seconds at 95 °C for DNA denaturation, 20 seconds at 60 °C for primer annealing and 10 seconds at 72 °C for primer extension) using a 7900HT Fast Real-Time PCR System (Applied Biosystems). Ct values were normalised against *Atpb5* (PrimerDesign, Southampton, UK) as previously described^6,21^ and fold changes (relative to control sample) were calculated for each gene according to the 2^−ΔΔCt^ method^54^.

### Protein extraction from mouse ovaries

Protein extraction from whole ovaries was performed after initial disruption with 25 G needles with Pierce IP lysis buffer (Thermo Fisher Scientific) containing protease inhibitors (cOmplete^TM^ Mini ULTRA tablets; Roche) and phosphatase inhibitors (PhosSTOP^TM^, Roche). Pooled ovaries (3) from day 4 and 16 mice were used for co-immunoprecipitation or (2) for processing cultured ovaries. Samples were vortexed, centrifuged and concentration of supernatant was measured using the Pierce bicinchoninic acid (BCA) protein assay kit (ThermoFisher). For subcellular fractionation, proteins were extracted from six day 4 mouse ovaries using a sequential lysis protocol as described previously^55^. This protocol utilises a gradient of detergent lysis buffers to sequentially yield a cytosolic fraction, a membrane-bound organelle fraction and a nuclear fraction, respectively. Equal amounts (3–5 µg) of the cytosolic, organelle and nuclear protein fractions were used for analysis by western blotting.

### Co-immunoprecipitation and Western blotting

Twenty-five µg of total protein was diluted with Pierce IP lysis buffer (ThermoFisher Scientific) and 2 µg of the immunoprecipitating antibody P27 (sc-528, Santa Cruz) were incubated for 1.5 hours at 4 °C with rotation. The same amount of protein was used to incubate with a non-specific IgG (Normal rabbit IgG I-1000, Vector Laboratories). 20 µl of Protein G magnetic beads (Merck-Millipore, MA, USA) were added to each aliquot and incubated with rotation for an additional hour. Bead/protein complexes were washed with IP buffer and gentle centrifugation. After the last wash, 35 µl of 5x loading buffer (EC-887, National Diagnostics, GA, USA) was used to elute the beads. Half of the volume of the IP samples was loaded on the western blot, and input samples (control for the IP) contained 20% of the initial amount of protein used for the IPs. Proteins were separated in 12% separating gel and 4% stacking PAGE gels. Proteins were transferred to a nitrocellulose membrane using a semi-dry transfer. Membranes were washed with TBS-Tween and blocked in either 5% (w/v) BSA (Sigma) for phospho-protein detection or 5% (w/v) non-fat dried milk at room temperature for 2 hours. Blots were washed in TBS-Tween before applying primary antibodies for CCND2 (0.4 µg/ml; sc-593, Santa Cruz), P27 (0.6 µg/ml; sc-528, Santa Cruz), p-SMAD3 (1:1000; ab52903, Abcam), p-AKT (1:1000; 4060, Cell Signaling), p-ERK1/2 (1:1000; 4370, Cell Signaling), p-S6 (1:1000; 2211, Cell Signaling) overnight at 4 °C. Antibodies were detected using HRP-conjugated secondary antibodies (1:20,000; P0448, DAKO) and ECL reagents (Westar Supernova enhanced chemiluminescent HRP substrate; Cyanagen, Bologna, Italy).

### Chromatin immunoprecipitation (ChIP) - qPCR

Whole ovaries were fixed with 1% formaldehyde (Sigma) in Leibovitz’s L-15 medium (Life Technologies) for 30 minutes. An EZ- Magna ChIP^TM^ G kit (Millipore) was used according to manufacturer’s instructions for the preparation of nuclear extracts and subsequent ChIP. 2% of the initial volume was taken as control for the IPs (Input). Protein G magnetic beads (Merck-Millipore) were added to each aliquot along with 1 µg of immunoprecipitating antibody against SMAD2/3 (sc-133098, Santa Cruz) and non-immune mouse IgG (I-200, Vector Laboratories). As a positive control, an antibody against the protein FOXL2 was used (0.2 µg/ml; sc-55655, Santa Cruz), since *Ccnd2* and *p27* are known targets of this protein^56,57^. An equal volume (4 µl) of IP sample, control or RNAse/DNAse free water (additional negative control) were analysed by qPCR in duplicate using primers designed to flank predicted FOXL2, SMAD3 and MYC binding sites (TRANSFAC; Table S1) in 384 well-plates as described above. Input, IPs and IgG control samples were analysed using the ‘% input’ method where the mean Ct value of each IP and IgG sample was normalised against the input sample. Since the starting input sample was 2% of the initial chromatin, a dilution factor of 50 or 5.64 cycles (log 2 of 50) was subtracted and calculations were performed as follows: i) Mean Ct INPUT – 5.64, ii) Mean Ct IP (or IgG) - Mean Ct INPUT = ΔCt, iii)% INPUT = (2 ^(−ΔCt)^) × 100.

### Statistical analysis

For immunofluorescent quantification of SMAD3, CCND2 and P27, follicle stage-specific differences in expression were analysed using a non-parametric Kruskal-Wallis test with a *post-hoc* Dunn’s multiple comparisons test. For all gene expression analyses by qPCR, fold changes between groups were evaluated using one-way ANOVAs with *post-hoc* Bonferroni multiple comparisons tests. For the ChIP-qPCR data, non-parametric Mann-Whitney U tests were used to compare % input values in SMAD3 vs IgG control groups. Western blot data was analysed using one-way ANOVAs with *post-hoc* Tukey’s multiple comparisons tests. Effect of different culture conditions on oocyte size was evaluated using a Kruskal-Wallis test with *post-hoc* Dunn’s multiple comparisons. All analyses were carried out using Prism (v7.03; Graphpad). Differences were considered significant when P < 0.05.

## Supplementary information

Supplemental figs and table

## Data Availability

All data generated or analysed during this study are included in this published article (and its Supplementary Information files).
