# Supplementary material for: SMAD3 directly regulates cell cycle genes to maintain arrest in granulosa cells of mouse primordial follicles
Source: Sci Rep. 2019 Apr 24;9:6513. doi: 10.1038/s41598-019-42878-4 (PMC6478827; doi:10.1038/s41598-019-42878-4)
Supplement: Supplementary file 1 — Supplemental figs and table [file 41598_2019_42878_MOESM1_ESM.docx]

**SMAD3 directly regulates cell cycle genes to maintain arrest in granulosa cells of mouse primordial follicles**

Sofia Granados-Aparici, Kate Hardy, Stephen Franks, Isam B. Sharum, Sarah L. Waite, and Mark A. Fenwick

Supplemental Figures S1-S7 and Table S1

**Supplemental Figure 1. Additional details from blots presented in Figure 1H (red box)**

Proteins were extracted from a pool of six-day 4 mouse ovaries using a sequential lysis protocol as described in the *materials and methods*. 3-5µg protein derived from the cytoplasmic, membrane (non-nuclear) and nuclear protein fractions were loaded alongside a protein size marker (ladder). Proteins were transferred to a nitrocellulose membrane, which was cut (black box shows approximate boundary of membrane) and probed for SMAD3 or GAPDH as described in the *materials and methods*. *Note:* as GAPDH is a cytoplasmic protein, it is not expected to be expressed in the nuclear fraction.

**Supplemental Figure 2. Additional details from blots presented in Figure 2I (red box)**

Proteins were extracted from a pool of three-day 4 (A,C) or three-day 16 (B,D) mouse ovaries and 25µg of each sample was incubated with anti-P27 IgG or non-specific IgG for immunoprecipitation as described in the *materials and methods*. Each gel was loaded with a sample of protein lysate without immunoprecipitation as a positive control (IN; 5µg), a P27 immunoprecipitated sample (IP; 12.5µg), a non-specific rabbit IgG sample (IgG; 12.5µg) and a protein size marker (ladder). Proteins were transferred to a nitrocellulose membrane and probed for CCND2 (A,B) or P27 (C,D) as described in the *materials and methods*. Red arrows indicate heavy and light Ig chains from the immunoprecipitated antibody.

**Supplemental Figure 3. Additional details from blots presented in Figure 3 (red boxes)**

Chromatin was isolated using ChIP from individual day 4 or day 16 ovaries using non-specific mouse IgG, SMAD2/3 IgG, or FOXL2 IgG as described in the *materials and methods*. An equal volume (4µl) DNA sample was amplified by PCR using primers designed to flank predicted FOXL2, SMAD3 and MYC binding sites. A positive sample of chromatin (Input) prior to immunoprecipitation is also shown. Note the FOXL2 and SMAD3 PCR products from the d4 *Ccnd2* gel have been switched for consistency of presentation. This is indicated by the divisional spacing in the d4 gel presented in Fig. 3A of the manuscript.

**Supplemental Figure 4. Additional details from blots presented in Figure 4C (red boxes)**

Proteins were extracted from cultured mouse ovaries (2 ovaries per sample) and 15µg lysate was loaded into each lane. Products were to a nitrocellulose membrane and probed for phospho-intermediates in the canonical TGFβ (p-SMAD3), PI3 kinase/Akt (p-AKT), p44/42 MAPK (p-ERK1/2) and mTOR (p-S6) pathways. C: control, T: TGFβ1 ligand, I: A83-01 inhibitor samples.

**Supplemental Figure 5. Classification of small follicles in the mouse ovary**

Follicles were classified according to the number of GC nuclei, as determined by DAPI staining (blue; upper panels; examples of individual GC nuclei labelled in yellow) and the area of the oocyte, which was facilitated by staining GCs with SMAD3 (red; lower panels; oocytes indicated with dotted line). Only follicles that had a clearly identifiable oocyte nucleus were included for analysis. Scale bar equals 25µm. Reference values are indicated in the table.


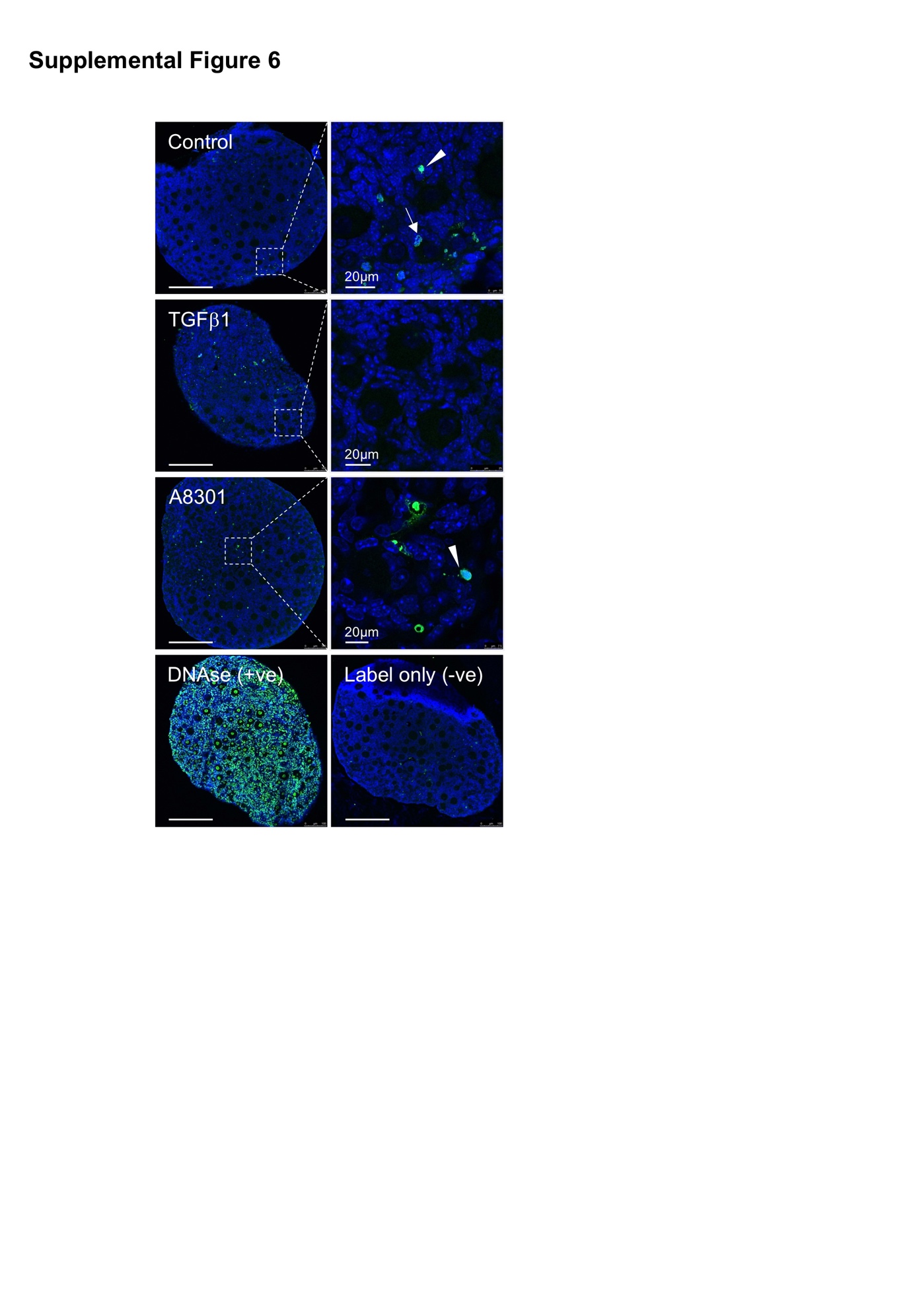


**Supplemental Figure 6. TUNEL labelling in cultured ovaries**

Whole neonatal mouse ovaries (d4) were maintained *in vitro* and exposed for 2 hours with either 10ng/ml of TGFβ1 ligand (TGFβ1), 1µM A83-01 inhibitor (A83-01), or 1µM DMSO (Control). Ovaries were then placed in basic culture media for two additional days prior to processing for staining as described in the *Materials and Methods*. In brief, following de-waxing, sections were washed in ice-cold permeabilization solution (0.1% w/v tri-sodium citrate, 0.1% (v/v) Triton X-100) before washing in PBS and applying TUNEL solution (10% (v/v) TUNEL enzyme, 90% (v/v) TUNEL FITC label (Roche)) for 1h at 37°C. One section was pre-incubated with DNase I (QIAGEN) as a positive control and one section was incubated with TUNEL label (no enzyme) as a negative. Sections were washed in PBS and mounted with Prolong Gold Antifade with DAPI (Invitrogen). Three sections from each group were stained and imaged using a Leica SP5 confocal microscope. A few TUNEL positive GCs (arrow) and stromal/interstitial cells (arrowhead) were detectable in each group. Scale bars equal 200µm unless stated.

**Supplemental Figure 7**

**Supplemental Figure 7. Quantification of immunofluorescence of SMAD3, CCND2 and P27 by follicle stage in d4 and d8 ovaries**

To visualise differences between measurements taken from d4 ovaries (red) and d8 ovaries (blue), total SMAD3 and nuclear SMAD3 in GCs has been re-plotted from raw data shown in Figure 1; nuclear CCND2, nuclear P27 and nuclear P27/CCND2 has been re-plotted from raw data shown in Figure 2. All data points represent median expression level ± interquartile ranges. No age-dependent differences were evident within each dataset (P>0.05; Kruskal-Wallis test). PF, primordial; T, transitional; P, primary; P+, primary plus, S, secondary follicle.

**Supplemental Table 1. Primers used for PCR assays.**

| **Gene** | **Sequence (5’🡪3’)** | **Product size (bp)** |
| --- | --- | --- |
|  |  |  |
| *Ccnd2^a^* | For: CTTGTAGCCCATTCAGACACAG  Rev: TATCATCCACGTGTGCTGTAGA | 165 |
| *Myc^a^* | For: AAATCCTGTACCTGGTCCGATT  Rev: CCACAGACACCACATCAATTTT | 184 |
| *P27^a^* | For: AGTCAGCGCAAGTGGAATTT  Rev: AGTAGAACTCGGGCAAGCTG | 100 |
| *Smad2^a^* | For: CGTCCATCTTGCCATTCAC  Rev: GTCCATTCTGCTCTCCACCA | 102 |
| *Smad3^a^* | For: GTCAAAGAACACCGATTCCA  Rev: TCAAGCCACCAGAACAGAAG | 154 |
| *Smad7^a^* | For: AGTCAAGAGGCTGTGTTGCTGT  Rev: CATTGGGTATCTGGAGTAAGGA | 130 |
| *Ccnd2^b,c^* | For: TTCTGCAGGAGGGTCATATTCT  Rev: AATGAGGAACAAGGAAAGGCTT | 207  (-652) |
| *Myc^b^* | For: CGAGATGGAGTGGCTGTTT  Rev: TGTGTGGAGTGATAGAGGGT | 207  (-659) |
| *Myc^c^* | For: GGCATATTCTCGCGTCTAGC  Rev: TGAAGACAAACGGATGAACAGT | 154  (-1072) |
| *P27^b,c^* | For: TTAGTGTCTGGGACGGCTCTAA  Rev: GACCCCAGAAGTCTCTGCTATG | 175  (-751) |

Superscripts indicate assay: ^a^ qPCR, ^b^ ChIP-PCR; ^c^ ChIP-qPCR

For ChIP-PCR/qPCR assays, all primer pairs align to proximal regions upstream of exon 1 as indicated by numbers in parentheses.
